# A Prospective Multicenter Cohort Surveillance Study of Invasive Aspergillosis in Patients with Hematologic Malignancies in Greece: Impact of the Revised EORTC/MSGERC 2020 Criteria

**DOI:** 10.3390/jof7010027

**Published:** 2021-01-05

**Authors:** Maria Siopi, Stamatis Karakatsanis, Christoforos Roumpakis, Konstantinos Korantanis, Helen Sambatakou, Nikolaos V. Sipsas, Panagiotis Tsirigotis, Maria Pagoni, Joseph Meletiadis

**Affiliations:** 1 Clinical Microbiology Laboratory, “Attikon” University General Hospital, Medical School, National and Kapodistrian University of Athens, 12462 Athens, Greece; marizasiopi@hotmail.com; 2 Department of Hematology and Lymphoma, Unit of Bone Marrow Transplantation, “Evangelismos” General Hospital, 10676 Athens, Greece; karakatsanisjstamatis@gmail.com (S.K.); marianpagoni@yahoo.com (M.P.); 3 2nd Department of Internal Medicine, Hematology Unit, “Attikon” University General Hospital, Medical School, National and Kapodistrian University of Athens, 12462 Athens, Greece; chrisroubak@gmail.com (C.R.); panagtsirigotis@gmail.com (P.T.); 4 Pathophysiology Department, “Laiko” General Hospital, Medical School, National and Kapodistrian University of Athens, 11527 Athens, Greece; konstantinos_korinthos26@yahoo.gr (K.K.); nsipsas@med.uoa.gr (N.V.S.); 5 2nd Department of Internal Medicine, “Hippokration” General Hospital, 11527 Athens, Greece; helensambatakou@msn.com

**Keywords:** invasive aspergillosis, hematology patients, diagnosis, Greece, 2020 EORTC/MSGERC criteria

## Abstract

Data concerning the incidence of invasive aspergillosis (IA) in high-risk patients in Greece are scarce, while the impact of the revised 2020 EORTC/MSGERC consensus criteria definitions on the reported incidence rate of IA remains unknown. A total of 93 adult hematology patients were screened for IA for six months in four tertiary care Greek hospitals. Serial serum specimens (*n* = 240) the sample was considered negative by PCR were collected twice-weekly and tested for galactomannan (GM) and *Aspergillus* DNA (PCR) detection. IA was defined according to both the 2008 EORTC/MSG and the 2020 EORTC/MSGERC consensus criteria. Based on the 2008 EORTC/MSG criteria, the incidence rates of probable and possible IA was 9/93 (10%) and 24/93 (26%), respectively, while no proven IA was documented. Acute myeloid leukemia was the most (67%) common underlying disease with most (82%) patients being on antifungal prophylaxis/treatment. Based on the new 2020 EORTC/MSGERC criteria, 2/9 (22%) of probable and 1/24 (4%) of possible cases should be reclassified as possible and probable, respectively. The episodes of probable IA were reduced by 33% when GM alone and 11% when GM + PCR were used as mycological criterion. The incidence rate of IA in hematology patients was 10%. Application of the 2020 EORTC/MSGERC updated criteria results in a reduction in the classification of probable IA particularly when PCR is not available.

## 1. Introduction

Over the last decades, advances in the treatment of hematologic malignancies have been paralleled by a growing prevalence and changing epidemiology of invasive aspergillosis (IA) in hematology patients [1,2,3]. The incidence rates of IA among this high-risk population are very much dependent on local epidemiology as they may vary according to patient characteristics and care practices [2,4,5], while they are even subject to seasonal variations of climate variables and local conditions [6]. It is noteworthy that the reported epidemiology of IA in patients with hematologic malignancies also depends on the local approach to diagnosing IA as more systemic testing and combination of multiple biomarkers to achieve adequate sensitivity have shown to increase its detection [7,8]. Furthermore, local monitoring is critical since the incidence pattern of IA may guide different management strategies (e.g., use of primary prophylaxis or not) [9], whereas it could contribute to the rationalization of excessive and not-targeted antifungal therapy use that may result in pronounced adverse effects, elevated medical costs, and emergence of resistance [10]. Hence, several experts recommend the need for heightened awareness and surveillance of IA in hospitals [11,12]. To date, the burden of IA among hematology patients in Greece remains obscure [13]. Of note, only a few hospital-based microbiological laboratories in Greece have currently reported diagnostic capacity for invasive fungal infections relying on serological and/or molecular assays, while having the capacity does not translate into routine testing due to lack of funding [14].

**Figure 1:**
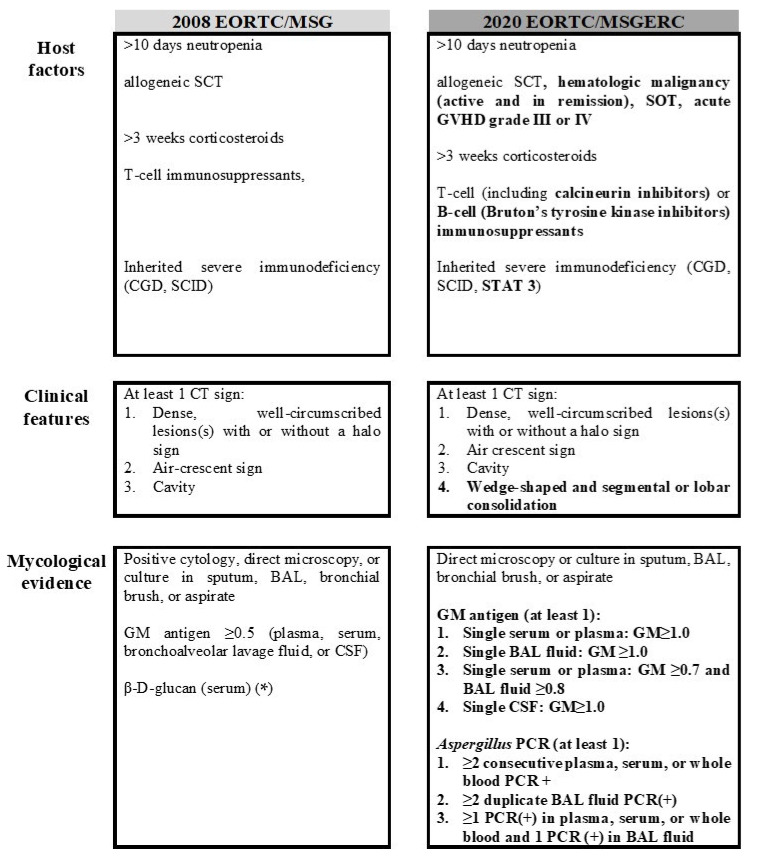
Criteria for probable invasive pulmonary aspergillosis according to the 2008 European Organization for Research and Treatment of Cancer-Invasive Fungal Infections Cooperative Group/National Institute of Allergy and Infectious Diseases Mycosis Study Group (EORTC/MSG) and the recently updated 2020 EORTC/MSGERC (revised conditions) definitions (differences between them are highlighted in bold). At least one host factor, a clinical feature and mycological evidence are required for probable invasive aspergillosis. Cases that meet the host and clinical criteria but not the mycological criterion are considered possible invasive fungal disease. SCT: stem cell transplantation, CGD: chronic granulomatous disease, SCID: Severe combined immunodeficiency, GVHD: graft versus host disease, CT: computer tomography, BAL: bronchoalveolar lavage fluid, GM: galactomannan, CSF: cerebrospinal fluid, PCR: polymerase chain reaction. (*) The β-D-glucan assay was included as a marker for probable invasive fungal diseases, because this test detects other species of fungi besides *Aspergillus*.

Timely diagnosis is the workhorse of the early initiation of targeted systemic antifungal therapy, which is vital for a successful clinical outcome in immunocompromised individuals with IA [15]. Nevertheless, its accurate diagnosis early enough to be of value in patient management is still challenging since nearly two-thirds of *Aspergillus* infections remain undiagnosed ante-mortem [16]. In 2008 the European Organization for Research and Treatment of Cancer-Invasive Fungal Infections Cooperative Group/National Institute of Allergy and Infectious Diseases Mycosis Study Group (EORTC/MSG) Consensus Group devised criteria for classification of potential cases according to the likelihood of underlying invasive fungal disease (IFD) into proven, probable, and possible [17]. These definitions were designed for research purposes and not for clinical decision making. Recently, these classification criteria have been revised by EORTC/MSG Education and Research Consortium (ERC) and separate criteria for defining probable IFD caused by specific pathogens have been provided [18]. The criteria of proven IFD were expanded to include amplification of fungal DNA by polymerase chain reaction (PCR) combined with DNA sequencing when molds are seen in formalin-fixed paraffin-embedded tissue together with microscopic detection and culture form sterile specimens. The designation of a probable IFD required the combination of a host factor, a clinical feature and a microbiological criterion (all three criteria should be fulfilled for probable IFD). Cases that met the host and clinical criteria but without mycological support were classified as possible IFD. Major changes in the 2020 updated definitions include the expansion of host factors to include active and in remission hematologic malignancies, solid organ transplantation, acute graft-versus-host disease grade III or IV and STAT 3 immunodeficiency, the addition in the radiographic features of the wedge-shaped and segmental or lobar consolidation and changes in the mycological criteria for IA (Figure 1). For the first time, PCR-based assays are included to help define probable IA, using various clinical specimens, while revised thresholds for galactomannan (GM) index replaced those of the manufacturer. Finally, the detection of 1,3-β-D-glucan is not considered to provide mycological evidence of any invasive mold disease [18].

Given the absence of data in a real-life cohort of hematologic malignancies and the impact of the new criteria on IA epidemiology, the aim of the present prospective, multicenter study was to describe the contemporary epidemiology of IA in hematology patients in Greece together with the underlying conditions, antifungal treatment and mycological diagnostic tests. In order to evaluate the impact of the revised EORTC/MSGERC definitions on the rate of the diagnosis of IA, we performed a comparative analysis defining the IA episodes by both the 2008 and the 2020 criteria.

## 2. Materials and Methods

Study design and population. A total of 93 adult patients with hematologic malignancies at risk for IA [19,20,21] according to the attending clinicians were screened for the detection of GM and *Aspergillus* DNA in serum samples collected during a 6-month period between 2013 and 2015 in each of four tertiary care hospitals in the area of Athens, Greece, namely “Attikon” University General Hospital (1 March–31 August 2013, *n* = 21), “Evangelismos” General Hospital (1 January−30 June 2014, *n* = 39), “Hippokration” General Hospital (1 June−30 November 2013, *n* = 12), and “Laiko” General Hospital (1 April−30 September 2015, *n* = 21). “Attikon” and “Evangelismos” operate state-wide autologous and allogeneic hematopoietic stem cell transplantation (HSCT) services, “Laiko” performs autologous HSCT only, and “Hippokration” has an inpatient oncology unit. 

Patient episodes (proven, probable, possible or no evidence of IA) were stratified according to both the 2008 EORTC/MSG [17] and the 2020 EORTC/MSGERC [18] definitions. Patients’ demographic (gender, age, body mass index (BMI), underlying disease) and clinical characteristics during the survey period (duration and degree of neutropenia (absolute neutrophil count <500/mm^3^), hepatic and renal function, current medications together with radiological, histological and microbiological findings, and outcome during hospitalization) were obtained from computerized databases of each center.

The study protocol was approved by the local institutional Review Board and Bioethics Committee of each participating hospital and written informed consent was obtained from each patient or relative.

Clinical samples. Serial serum specimens from all patients were collected twice weekly. The number of evaluable serum samples for the detection of circulating fungal biomarkers was 240. For most of patients there was one sample before neutropenia and several samples (3–15) during neutropenia. The obtained sera were stored at −70 °C until analyzed. A commercially available sandwich enzyme-linked immunoassay (Platelia *Aspergillus* EIA; Bio-Rad Laboratories) was used to quantify GM antigen in accordance with the manufacturer’s instructions. A result was considered positive when the index value was ≥0.5 [22]. A real-time PCR was developed in line with the published European *Aspergillus* PCR Initiative recommendations for serum [23]. *Aspergillus* DNA was extracted from 1 mL serum after enzymatic (incubation with protease K at 56 °C for 10 min) and mechanical (15 min vortex with glass beads) pre-treatment using the High Pure Viral Nucleic Acid Large Volume Kit (Roche, Athens, Greece) according to the manufacturer’s instructions. Real-time PCR was performed with a previously validated assay (2Asp assay) using *Aspergillus*-specific primers (ASF1 and ADR1) and probe (ASP28P) [24]. When no amplification was observed after 43 PCR cycles (*C*t), the sample was considered negative by PCR [24].

Data analysis. Median and interquartile ranges (IQR) were calculated for continuous variables, while numbers and percentages were calculated for categorical parameters. Categorical variables were compared between independent groups using chi-square and Fisher’s exact test and continuous variables were compared using one-way ANOVA. In any case, a two-tailed *p*-value of <0.05 was considered to reveal a statistically significant difference. Agreement between classifications based on 2008 and 2020 criteria was assessed with the kappa statistic. All data were analyzed using the statistics software package GraphPad Prism, version 7.0, for Windows (GraphPad Software, San Diego, CA, USA) and JMP7 (SAS Institute Inc., Cary, NC, USA).

## 3. Results

### 3.1. Patients’ Characteristics

Patients’ characteristics are displayed in Table 1. Overall, 48/93 (52%) hematology patients enrolled in the study were men of median (range, IQR) age 51 (18–83, 27) years. The underlying disease was acute myelogenous leukemia (AML) in 62 (67%) patients, acute lymphoblastic leukemia (ALL) in 12 (13%), myelodysplastic syndrome (MDS) in 5 (5%), non-Hodgkin’s lymphoma (NHL) in 2 (2%), and various other conditions in the remaining 12 (13%). Among patients, 22/93 (24%; 14 AML, 4 ALL, 2 Hodgkin disease, 1 NHL and 1 chronic lymphocytic leukemia) have undergone autologous HSCT. None of the patients received allogeneic HSCT. The crude mortality rate within hospital stay was 12% (11/93; 7 AML (1/7 autologous HSCT), 2 MDS and 2 other), either due to the underlying malignancy and/or due to multiple infections/sepsis (Table 1).

### 3.2. Antifungal Treatment

Most of the patients (82%, 76/93) had received ≥2 defined daily doses of antifungal drugs with 42% (32/76) receiving mold-active antifungal prophylaxis or treatment at the time serum samples were collected (Table 1). In particular, among the nine patients with probable IA all (100%) were treated with antifungal drugs; six received voriconazole, two micafungin and one echinocandin-voriconazole combination. Among the 24 cases of possible IA, 16 (67%) were treated with antifungal drugs; 2 received micafungin, 12 voriconazole, 2 liposomal amphotericin B. Among the 60 cases with no IA, 51 (85%) were treated with antifungal drugs with 12% (9/51) receiving mold-active agents. Of note, when routine therapeutic drug monitoring was performed during initiation of treatment/prophylaxis in 11/17 patients receiving 400 mg/day intravenous voriconazole [25], steady-state trough levels were on-target (2–6 mg/L) in 8/11 (72%) patients (mean (range) 3.4 mg/L (2.1–5.2 mg/L)) with the rest of patients (2 with probable and 1 with possible IA) having sub-therapeutic concentrations (mean (range) 1.0 mg/L (0.7–1.2 mg/L)) [9]. 

### 3.3. Episodes of Invasive Aspergillosis

According to the 2008 EORTC/MSG criteria where at least one positive GM result is required (GM index ≥0.5) as mycological evidence [17], there were 9/93 (10%) patients with probable IA and 24/93 (26%) cases classified as possible IA, while no proven IA was documented (Table 1). Of note, patients with probable IA had a median (range, IQR) GM index value of 1.05 (0.51–3.02, 0.76), of which 3/9 (33%) had a GM index 0.5–1 and 2/9 (22%) had serial positive GM results. The median (range) rates of probable and possible IA were 9% (0–19%) and 29% (15–48%), respectively, among the four participating centers. 

By applying the 2020 EORTC/MSGERC revised criteria, 2/3 cases with GM index 0.5–1 were downgraded from probable infection to possible, while 1/3 remained as probable taking into account the *Aspergillus* PCR, where ≥2 consecutive positive tests are required [18]. Moreover, re-classifying the GM negative episodes using the PCR test yielded four non-classifiable (host without clinical criteria) and one possible IA cases (host and clinical criteria) that now fulfilled the PCR microbiological criterion, with the latter being reclassified as probable. Overall, there were 8/93 (9%) cases of probable IA (11% reduction in the classification) and 25/93 (27%) cases of possible IA (4% increase in the classification) when the 2020 definitions were applied. Notably, only 2/8 (25%) patients with probable IA had both a GM index ≥1 and ≥2 consecutive PCR tests positive indicating that there is no significant overlapping between these two biomarkers, while a large number of GM negative patients (16/84; 19%) had only one PCR test positive but only 6% (5/84) had ≥2 consecutive PCR tests positive. The agreement between the classifications based on the two criteria was 97% (kappa statistic = 0.94).

The classification of IA episodes did not differ according to the patients’ demographics (gender (*p*  =  0.28), age (*p*  =  0.28), weight (*p*  =  0.21) and BMI (*p*  =  0.07)) and clinical parameters (type of underlying hematologic malignancy (*p*  =  0.37), autologous HSCT (*p*  =  0.55), and crude mortality (*p*  =  0.69)).

**Table 1:** Demographic and clinical parameters of the studied cohort. Patient episodes were stratified according to the 2008 EORTC/MSG criteria [17].

Patients’ Characteristics		Total (*n* = 93)	Probable IA (*n* = 9)	Possible IA (*n* = 24)	No IA (*n* = 60)
Sex	Male	48 (52%)	5 (56%)	9 (38%)	34 (57%)
	Female	45 (48%)	4 (44%)	15 (62%)	26 (43%)
Age (y) (median (range, IQR))		51 (18–83, 27)	44 (20–75, 17)	56 (37–79, 17)	50 (18–83, 27)
Weight (kg) (median (range, IQR))		70 (48–115, 18)	60 (57–77, 14)	65 (48–110, 14)	72 (51–115, 15)
BMI (kg/m^2^) (median (range, IQR))		24 (17–38, 5)	23 (19–24, 3)	23 (17–34, 4)	26 (20–38, 5)
Underlying hematologic malignancy	AML	62 (67%)	5 (56%)	19 (80%)	38 (63%)
	ALL	12 (13%)	3 (33%)	1 (4%)	8 (13%)
	MDS	5 (5%)	1 (11%)	1 (4%)	3 (5%)
	NHL	2 (2%)	0 (0%)	1 (4%)	1 (2%)
	Other ^a^	12 (13%)	0 (0%)	2 (8%)	10 (17%)
AutoHSCT		22 (24%)	1 (11%)	5 (21%)	16 (27%)
Antifungal therapy	Any	76/93 (82%)	9/9 (100%)	16/24 (67%)	51/60 (85%)
	L-AMB	3/76 (4%)	0 (0%)	2/16 (12.5%)	1/51 (2%)
	VRC	18/76 (24%)	6/9 (67%)	12/16 (75%)	0 (0%)
	POS	5/76 (7%)	0 (0%)	0 (0%)	5/51 (10%)
	ITC	3/76 (4%)	0 (0%)	0 (0%)	3/51 (6%)
	FLC	11/76 (14%)	0 (0%)	0 (0%)	11/51 (21%)
	CAS	7/76 (9%)	0 (0%)	0 (0%)	7/51 (14%)
	MFG	28/76 (37%)	2/9 (22%)	2/16 (12.5%)	24/51 (47%)
	VRC + CAS	1/76 (1%)	1/9 (11%)	0 (0%)	0 (0%)
Mycological criteria	GM index ≥ 0.5	9 (10%)	9 (100%)	0 (0%)	0 (0%)
	GM index ≥ 1	6 (6%)	6 (67%)	0 (0%)	0 (0%)
	1 PCR+ test	19 (20%)	3 (33%)	6 (25%)	10 (17%)
	≥2 PCR+ tests	8 (9%)	3 (33%)	1 (4%)	4 (7%)
Crude hospital mortality		11 (12%)	1 (11%)	4 (17%)	6 (10%)

^a^ Myeloma, chronic lymphocytic leukemia, chronic myeloid leukemia, Burkitt lymphoma, Hodgkin disease. Abbreviations. IA: invasive aspergillosis, IQR: interquartile range, BMI: body mass index, AML: acute myeloid leukemia, ALL: acute lymphoblastic leukemia, MDS: myelodysplastic syndrome, NHL: non-Hodgkin’s lymphoma, HSCT: hematopoietic stem cell transplantation, L-AMB: liposomal amphotericin B, VRC: voriconazole, POS: posaconazole, ITC: itraconazole, FLC: fluconazole, CAS: caspofungin, MFG: micafungin, GM: galactomannan, PCR: polymerase chain reaction.

## 4. Discussion

This is the first multicenter cohort study assessing the incidence of IA in high-risk hematology patients treated at Greek hematology/oncology centers. The incidence rate of probable IA was 10%, with AML being the most represented underlying disease, while no proven IA was documented. By applying the 2020 EORTC/MSGERC criteria, a small reduction (11%) in the number of cases classified as probable IA was observed. Our findings show that the key contributing factor to this reduction in the classification is the adaption of the increased by twofold GM index cut-off value from 0.5 to 1, which was partially compensated by the incorporation of *Aspergillus* PCR in the mycological criteria (≥2 consecutive PCR+ tests). Therefore, the revised EORTC/MSGERC definitions may not have a significant impact on the degree of the diagnostic certainty of IA in patients with hematologic malignancies providing that serial PCR data are available. Of note, without the PCR data, a 33% reduction in the classification of probable IA was observed using the GM as the only mycological criterion.

Invasive mold infections represent a significant challenge in the management of hematology patients. Given the changing face of their reported epidemiology due to new chemotherapeutic regimens and comorbidities, there is a continuous need to revisit their burden and trends capturing the era of evolving patterns in risk factors [26,27] and introduction of new therapies to treat hematologic malignancies [28,29]. While several studies have been conducted globally to estimate local IA incidence rates, similar data with the ultimate goal of the surveillance of the disease in high-risk patients in Greece do not exist. In a first attempt to depict the burden of serious fungal infections in Greece, the incidence of IA in non-ICU immunosuppressed hematology and solid organ-transplanted patients was estimated at 0.8/100.000 population (85 cases/year) [13]. Of note, in a recent survey among 141 physicians in the 26 Greek hospitals that serve patients with hematologic malignancies, only a minority reported capacity for serological (GM 53%, 1,3-β-d-glucan 13%) and molecular (*Aspergillus* PCR 7%) tests [14]. Therefore, IA surveillance in our country is challenging given the absence of consistent laboratory diagnostic tests. As a consequence of the unexpectedly high incidence of IA previously reported in a center in Greece [30], we carried out the first multicenter study investigating the epidemiology of IA in high-risk hematology patients. According to our findings, the incidence rate of probable IA was 10%, which was comparable with those previously reported for mixed populations of hematology patients on antifungal prophylaxis (4–17%, mean 7.6%) [31]. However, the latter meta-analysis indicated that the incidence rates of IA in hematology patients during remission-induction therapy was lower in patients on prophylaxis compared to those without prophylaxis (5.7% versus 11.1%, respectively). Since most of our patients were on antifungal prophylaxis/treatment, the true incidence rate of probable IA in the present study may be higher. Among 22 cases of autologous HSCT in our study, only one (4.5%) patient developed probable IA. In fact, since the duration of neutropenia after autologous HSCT is shorter, the incidence of IA is expected to be low, as previously reported (0.5–6%) [20].

To date, sufficient data on the contemporary epidemiology of IA in Greek hematology patients are lacking. During a 1.5-year (01/2014–05/2015) single-center observational study, Apostolidi et al. reported an incidence rate of 45.4% (proven plus probable IA) and a total cumulative incidence of 3.2 cases/100 patients with hematologic malignancies [30]. This high rate was explained due to bad hospitalization conditions in an old hospital building and is verified in the present study since the same center had the highest incidence rate of probable IA although lower than the previous report (19% versus 41%) [30]. One should notice, however, that only 17.6% (12/68) of patients received anti-mold prophylactic antifungal therapy in the latter study, as opposed in our study, where 42% (32/76) of patients were on anti-mold prophylaxis/treatment. This may explain the lower crude mortality rates found in the present study compared to the previous report (12% versus 26%) [30]. Notably, therapeutic drug monitoring is strongly or marginally recommended for hematology patients receiving posaconazole suspension or any form of voriconazole, respectively, for IA primary prophylaxis [9], while it should be kept in mind that the use of prophylaxis should certainly reduce the incidence of IFDs but it may also hamper their detection when there are breakthrough infections.

The fact that crude mortality rates were similar between patients with and without IA indicates that the rate of IA-attributable mortality is also low due to extensive use of antifungal drugs. The most common drug used in patients with probable (67%) or possible (75%) IA was voriconazole whereas in patients with no IA was micafungin (47%) or fluconazole (21%) indicating that despite the absence of the results of diagnostic tests when treatment was initiated, most patients with IA received correct treatment (78% with probable IA were treated with voriconazole or voriconazole + caspofungin and 88% with possible IA were treated with voriconazole or liposomal amphotericin B) based on clinical and host criteria. However, an alarming 22% of patients with probable IA and 12.5% with possible IA were not treated with the optimal therapy since they received micafungin. Furthermore, among the patients treated with voriconazole, we detected sub-therapeutic voriconazole steady-state trough levels at initiation of antifungal therapy in 28% of the patients, which is in line with previously reported data [32,33]. Voriconazole therapy may be further challenged by azole resistance as environmental azole-resistant *A. fumigatus* isolates have been found in Greece [34]. Of note, one-half patients with probable IA and off-target voriconazole concentration (0.7 mg/L) died during hospitalization. Thus, the percentage of patients with probable IA treated inadequately (micafungin + subtherapeutic voriconazole) were 50% emphasizing the importance of diagnostic tests and therapeutic drug monitoring.

In accordance with all major international guidelines [9,35], mold-active prophylaxis strategy has now been adopted in many centers for certain groups of hematology patients. Nevertheless, the administration of antifungal prophylaxis/treatment reduces the sensitivity of GM testing for IA [36,37,38] underestimating the true incidence of IA although recent controlled studies of posaconazole antifungal prophylaxis does not confirm this conclusion [39]. Based on these grounds and given the 2020 EORTC/MSGERC revised criteria, where the serum/plasma GM index cut-off increased from ≥0.5 to ≥1 in absence of GM levels in bronchoalveolar lavage fluid [18], the incidence of probable IA is expected to decrease in this particular setting. Indeed, our findings revealed a 33% reduction in the number of episodes stratified as probable IA based only on GM. When PCR data were also used, the reduction in the classification of possible IA based on the new criteria was reduced just by 11%. Thus, a significant reduction in incidence of possible IA is expected for centers utilizing the new EORTC/MSGERC criteria when PCR is not available. This finding may have a profound effect on diagnosis and treatment of IA given the limited *Aspergillus* PCR testing capacity (6–20%) that has been recently reported by diagnostic mycology laboratories worldwide [14,40,41,42].

Similarly, the biggest hurdle to broad implementation of *Aspergillus* PCR from blood specimens is its poor diagnostic performance in patients on systemic mold-active prophylaxis/treatment [43]. A large proportion of patients (19%) with possible or no evidence of IA had a single positive PCR test, which is in accordance with previous reports [44], justifying the use of PCR positivity for *Aspergillus* DNA in ≥2 serial blood samples as mycological criterion of IA in the 2020 EORTC/MSGERC updated criteria [18]. In fact, only 4% of cases classified as possible IA by the 2008 EORTC/MSG definitions were upgraded to probable infection as a result of the 2020 revision introducing PCR positivity in ≥2 serial samples as mycological evidence [18]. Interestingly, although 4/60 (7%) patients with no evidence of IA according to the 2008 criteria fulfilled the microbiological criterion for IA when the PCR test was used for re-classifying the episodes, they were still considered as non-classifiable cases due to the absence of clinical features. This group needs special attention because the type of immunosuppression in patients with hematologic malignancies affects the appearance of the radiologic presentation of IA [45], with a significant proportion (~40%) of them having small-airway lesions on their computer tomography scan that are not part of the current diagnostic criteria [46]. Given the impact of the 2020 criteria as per our data and acknowledging the fact that the total number of probable IA cases is not large enough to draw any firm conclusions, it appears that the diagnostic quality of the revised definitions is still heavily dependent on various influencing variables and therefore varies between settings.

## 5. Conclusions

An infection with an evolving epidemiology, such as IA, necessitates a local surveillance system given the inter-center variability in incidence rates and treatment modalities. The incidence of probable IA in the present study was 10% although a significant inter-center variation was observed and most patients were on antifungal prophylaxis/treatment. In-depth knowledge of the problem will help to design appropriate treatment strategies as shown in the present study where the employment of antifungal prophylaxis in a center with high incidence rate of IA had an impact on mortality. Clearly, a major challenge of IA in hematology patients remains making a definitive diagnosis. The implementation of the 2020 EORTC/MSGERC revised criteria resulted in reduction of the reported incidence of probable IA by 33% when the GM alone and 11% when the combination of GM and PCR were used as mycological evidence. The EORTC/MSGERC definitions were not designed to be used in routine clinical practice and there is certainly a need for new accurate assays to enable the diagnosis of the infection, ideally at an early stage.

## Data Availability

Data available on request.
